# Asymmetric conformation of the high-spin state of iron(II)-tris(2,2-bipyridine): Time-resolved x-ray absorption and ultraviolet circular dichroism

**DOI:** 10.1063/4.0000268

**Published:** 2024-11-08

**Authors:** Nico Sanna, Costantino Zazza, Giovanni Chillemi, Elisabetta Pace, Francesco Cappelluti, Luigi Bencivenni, Malte Oppermann, Maurizio Benfatto, Majed Chergui

**Affiliations:** 1 Department for Innovation in Biological Agro-food and Forest systems (DIBAF), University of Tuscia, Largo dell' Università snc, 01100 Viterbo, Italy; 2 Institut of Biomembranes, Bioenergetics and Molecular Biotechnologies, Consiglio Nazionale delle Ricerche, 70126 Bari, Italy; 3 Laboratori Nazionali di Frascati – INFN, Via E. Fermi 44, 00044 Frascati, Italy; 4 Department of Physics and Astronomy, University of Aarhus, Ny Munkegade 120, 8000 Aarhus C, Denmark; 5 Department of Chemistry, Sapienza University, P. le A. Moro 5, 00185 Rome, Italy; 6 Lausanne Centre for Ultrafast Science (LACUS), Ecole Polytechnique Fédérale de Lausanne, ISIC, FSB, Station 6, CH-1015 Lausanne, Switzerland; 7 Departement Chemie, Universität Basel, St. Johanns-Ring 19, 4056 Basel, Switzerland; 8 Elettra - Sincrotrone Trieste S.C.p.A., S.S.14 Km.163, 5 in Area Science Park, I - 34149 Trieste, Italy

## Abstract

We analyze the structures of the low-spin (LS) ground state and the high-spin (HS) lowest excited state of the iron-(II)-tris bipyridine complex ([Fe(bpy)_3_]^2+^) using density functional theory PBE methods, modeling the solvent interactions with conductor-like polarizable continuum model. These calculations are globally benchmarked against a wide range of experimental observables that include ultraviolet-visible linear absorption and circular dichroism (CD) spectra and Fe K-edge x-ray absorption near edge spectra (XANES). The calculations confirm the already established D_3_ geometry of the LS state, as well as a departure from this geometry for the HS state, with the appearance of inequivalent Fe–N bond elongations. The simulated structures nicely reproduce the above-mentioned experimental observables. We also calculate the vibrational modes of the LS and HS states. For the former, they reproduce well the vibrational frequencies from published infrared and Raman data, while for the latter, they predict very well the low-frequency vibrational coherences, attributed to Fe-N stretch modes, which were reported in ultrafast spectroscopic experiments. We further present calculations of the high-frequency region, which agree with recent ultrafast transient infrared spectroscopy studies. This work offers a common basis to the structural information encoded in the excited state CD and the Fe K XANES of the HS state tying together different structural IR, UV-visible, and x-ray observables.

## I. INTRODUCTION

Spin crossover (SCO) complexes are molecules containing transition metals, most frequently Fe(II) ions, which undergo a ΔS = 2 change of spin from the singlet low-spin (LS, S = 0) ^1^A_1_ ground state to the lowest high-spin ^5^T_2_ (HS, S = 2) quintet excited state under the effect of external stimuli such as temperature, pressure, light and/or engineering of the ligands.^1,2^ This makes them attractive for several applications, in particular, magnetic data storage. The discovery of light-induced spin state trapping (LIESST) at cryogenic temperatures in 1984 represented a significant development in this respect,^3^ with some complexes reaching HS state lifetimes of several days depending on the organic ligand and the temperature. In the near-octahedral ligand field of Fe(II) SCO complexes, the d-orbitals split into lower t_2g_ bonding and upper antibonding orbitals. In the ground state, all six electrons are in the former while in the HS state, two of these end up in the upper e.g., orbitals leading to an elongation of the Fe-N bond length by about 0.2 Å, which is nearly identical throughout the family of Fe(II)-polypyridine complexes.^4,5^

In recent years, Fe(II)-tris-bipyridine ([Fe(bpy)_3_]^2+^) has emerged as the prototypical system for the description of light-induced SCO dynamics. Indeed, the relatively high energy difference between the LS and HS states leaves photoexcitation as the only stimulus for SCO. In the visible and ultraviolet, photoexcitation leads to rapid decay with unity quantum yield of the electronic states to the lowest electronic state, which is the metastable high-spin state (HS, S = 2).^1,6–18^ Visible excitation of the singlet metal-to-ligand-charge-transfer (^1^MLCT) state is more commonly used. Thereafter, decay occurs via the intermediate ^3^MLCT^7^ and the metal-centered (MC) states^13^ into the HS ^5^T_2_ state. Various theoretical interpretations have been proposed for the mechanism and the pathway(s) leading from the LS to the HS state.^19–25^ At room temperature and in an aqueous environment, the back-SCO proceeds via non-radiative HS state decay into the LS ground state in ∼0.6 ns.^5,26^ This is a relatively fast decay considering the ΔS = 2 character of the transition and it is indeed the shortest for the entire family of Fe(II) polypyridine complexes.

The HS-LS back-SCO in Fe(II)-polypyridine complexes has been rationalized in the framework of the energy gap law^2,27^ assuming a single reaction coordinate (the Fe-N bond) characterized by a symmetric displacement of all Fe-N bonds, and a second order spin–orbit coupling matrix element between the HS and LS states. In this picture, the low-temperature relaxation is attributed to tunneling from the HS to the LS states due to their largely different Fe-N equilibrium distances. However, already 40 years ago, theoretical works suggested that in addition to the Fe-N radial expansion, twisting or torsional distortions may also play a role in the back-SCO mechanism of unconstrained tris-chelate complexes, such as [Fe(bpy)_3_]^2+^, in the sense that the entropy-driven racemization of enantiopure or enantioenriched solutions at room temperature (RT) is accompanied by a change of spin-state.^28–35^ In particular, low-frequency torsional twisting modes associated with racemization result in a surface crossing of the ^1^A_1_ and ^5^T_2_ states, while stabilizing the ^3^T_1_ state and thus increasing the associated spin–orbit coupling term. In recent years, theoretical studies of the SCO have been carried out by density functional theory (DFT) (e.g., see Ref. 36 and references therein) often comparing the iron atom with chemically similar metals (for instance Ru)^37^ or by time-dependent DFT (TD-DFT).^19–21^,^38–40^ However, several of these approaches relied on the assumption of a symmetric radial displacement of all N atoms along the Fe-N bonds, which is considered to be accompanied by substantial torsional motion, converting the molecule between an octahedral structure (D_3_) and a trigonal-prismatic one (D_3h_ or C_2v_) from the LS to the HS states.^34^

Regarding the geometric structure of the LS state of [Fe(bpy)_3_]^2+^, x-ray diffraction^41^ and x-ray absorption spectroscopy (XANES and EXAFS) data are available,^5,42^ complemented by infrared^37^ and Raman^43,44^ spectra. For the HS state, XANES and EXAFS data are available,^5,42^ as well as low-frequency vibrational wave packet dynamics.^8,12,15,17,18^ Very recently, ultrafast transient IR spectra of the HS have also been reported.^45^ However, it should be mentioned that for several Fe(II)-polypyridine SCO systems with somewhat different ligands, there is a rich literature of steady-state and time-resolved IR and Raman spectroscopy whose information content can, to a certain extent, be applied to [Fe(bpy)_3_]^2+^.^26,43–48^

Ultrafast optical spectroscopy studies showed that the HS state is reached in ≪100 fs, upon excitation of the ^1^MLCT state, often yielding a rich pattern of wave packet oscillations in the HS state.^8,9,13,15^ This trend (ultrafast SCO and generation of wave packets in the HS state surface) is not specific to [Fe(bpy)_3_]^2+^ but was observed in other Fe(II) SCO complexes,^18,49^ including non-polypyridine ones.^50–54^ Most of these studies reported observation of low-frequency modes, which are very important since they concern the Fe–ligand bonds. However, it is not to be excluded that higher frequency modes could coherently be excited had a higher temporal resolution been used in the above-cited experiments.

Although well-documented theoretically,^22,28,30–32,35^ experimental evidence for the above-mentioned torsional distortions is scarce.^35^ Ideally, this would be provided by a structural analysis of the HS state. X ray diffraction has largely been used in the case of pressure-, temperature- and light-induced SCO^4^ complexes that can be crystallized and in which the HS state is long-lived,^50,51^ but the torsional distortion was not reported, probably due to limited resolution. The non-symmetric HS geometry was attributed by Pàpai *et al.* to Jahn–Teller (JT) effects arising from the degenerate electronic configuration of the quintet state multiplicity due to the occupation of two e_g_^*^ type antibonding orbitals. Symmetry breaking is not only due to JT distortions;^22^ *ab initio* molecular dynamics studies of an aqueous solution of [Fe(bpy)_3_]^2+^ with counterions show that solute–solvent interactions—with water molecules intercalated between the bpy ligands^24,55^—might play a key role in modulating the conformation of such an organometallic compound at room temperature.

In a recent article, Oppermann *et al.* combined ultrafast transient absorption, transient anisotropy and transient circular dichroism (CD) spectroscopy to the study of photoexcited enantiomers of a di-methylated form of the [Fe(bpy)_3_]^2+^complex (denoted [Fe(dm-bpy)_3_]^2+^).^56^ To our knowledge, this work established, for the first time, an experimental verification of the above-mentioned decade long predictions of a departure of the HS state from the D_3_ symmetry. Indeed, the transient CD results show that the HS state adopts the so-called Ray–Dutt twisting coordinate,^34^ leading to an enhanced spin–orbit coupling of the HS state with the LS ground state, thus facilitating its non-radiative decay and here, we model the structural information on the HS state based on the transient CD data.

For a short-lived HS SCO complexes such as [Fe(bpy)_3_]^2+^, time-resolved x-ray^57^ and electron^58^ diffraction have been implemented, but aside from being in crystals, the structural analysis of the HS state assumed a symmetric displacement of the ligands. For solutions, time-resolved x-ray absorption spectroscopy (XAS)^59,60^ is the structural tool of choice, as it delivers detailed short-range structural information via the analysis of the x-ray near-edge absorption structure (XANES)^61^ and the extended x-ray absorption fine structure (EXAFS).^42,62^ In time-resolved XAS, the measured signal is the difference between the absorption of the sample after and before photoexcitation.^59^

Here we computationally investigate the molecular structure of the [Fe(bpy)_3_]^2+^ complex in the LS and HS states by identifying the stationary points of the geometry landscape through the study of *ab initio* molecular vibrations by normal mode analysis within the QM-DFT approach *in vacuo* and in the solvent, using the popular Conductor-like Polarizable Continuum Model (C-PCM) method.^63^ These methods confirm the already observed trends that while the LS state is indeed a symmetric D_3_ structure, the HS state exhibits non-symmetric Fe–N distortions leading to a C_1_ symmetry. These conclusions are supported by different experimental observables, namely,: the steady-state and time-resolved UV-visible absorption spectra of [Fe(bpy)_3_]^2+^ in water,^8,15^ its steady-state and time-resolved XANES spectra,^5,42,64^ and the steady-state and time-resolved CD spectra.^56^ Further to this, we calculated the vibrational modes and spectra of the system, which we benchmark in the low-frequency vibrational modes against the vibrational wave packets observed in the HS state,^8,15,17,18,45^ while the high frequency modes are calculated and benchmarked against transient IR data in Ref. 45.

## II. METHODS

### A. Quantum mechanical calculations

All quantum mechanical (QM) computations were performed *in vacuo* and with the Conductor-like Polarizable Continuum Model (C-PCM)^47^ method using the Gaussian package.^65^ The work by Daku *et al.*^36^ was a seed to investigate the performance of density functional theory (DFT) methods for computing reference molecular geometries for the fitting of the XANES spectra. To this end, considering the electronic states investigated there and starting from x-ray crystallography^41^ and the XANES fitted data^5^ the molecular geometry of [Fe(bpy)_3_)]^2+^ was extracted and its symmetric D_3_ structure preliminarily optimized at the BHLYP/6–311+G^**^ level for the ^1^A_1_ singlet spin (Low-Spin – LS) ground state. A comprehensive refinement of the ground state molecular structure computed using the hybrid BHLYP^66,67^ was then performed by adopting the same 6–311+G^**^ basis set using the GGA (generalized gradient approximation) PBE^68^ functional with the inclusion (10%) of the exact (Hartree−Fock) exchange (hereafter termed as PBE^*^). This choice was actually driven by results presented by Daku *et al.*;^36^ in particular the authors concluded that hybrid functionals tend to give longer metal–ligand bond lengths than the GGAs, and that the more important the exact-exchange contribution the longer the metal–ligand bond. The admixture of 25% exact-exchange to the PBE functional—as in the PBE1PBE formulation—was reported to be in connection with an increase in the Fe–N distance by 0.022 (from 1.971 to 1.993 Å with 6–311+G(d,p) atomic basis set). For this reason, always remaining in the field of the hybrid and GGA functionals for numerical reasons, we have thought to introduce in our work and for the first time, a relatively small fraction (10%) of the exact-exchange in the PBE functional. Such a sort of “contamination” while resembling that already applied in the case of the B3LYP functional (termed as B3LYP^*^ in the literature)^36^ allow us to better reproduce the UV/Vis absorption signals in the region between 350 and 450 nm in gas-phase. The same procedure was adopted for the HS ^5^T_2_ open-shell excited state to investigate its lowest energy conformation treated in an unrestricted Kohn–Sham (KS) approach, after releasing the constraint of the D_3_ molecular symmetry. The calculations were performed using an *ultrafine* grid of Gaussian, that is 99 590 points, for the integral calculation and to minimize the numerical noise, the “VeryTight” criterion was adopted for all geometry optimization calculations (RMS force threshold set to 10^−6^ Hartrees/in bohr). All stationary points characterized by calculations of the first and second derivatives were found to be a local minimum at the best level of method adopted, namely, PBE^*^/6–311+G^**^ while a further energy refinement was carried out at PBE^*^/6–311++G(3df,3pd)//PBE^*^/6–311+G^**^ level of calculation. On the optimized geometries, restrained electrostatic potential (RESP)^69^ charges were calculated using the AMBER package for all atoms with optimized *ab initio* distances as equilibrium values.^66^

The UV-Visible (200–700 nm) absorption and circular dichroism (CD) spectra were computed using the time-dependent DFT^70^ and within the continuum polarizable PCM methods by re-optimizing the gas phase molecular geometry in water, dichloromethane (DCM) and trichloromethane (TCM), using the same functional and basis set indicated so far, with a number of transition states from 100 for the LS state to 150 for the HS state to compute their absorbances. In order to compare computed spectra with the experimental ones, calculated oscillator strengths were convoluted with Gaussian functions and computed with the aid of GaussSum^71^ code with a full-width half-maximum (FWHM) of 0.40 eV.

### B. Simulations of the XANES spectra

The MXAN software procedure employs the full multiple scattering (MS) approach within the muffin-tin approximation for the shape of the potential to calculate the XANES part of the x-ray absorption cross section. The total charge density needed to calculate the whole potential is obtained as the superposition of the charge density of each single atomic species obtained through a self-consistent Dirac–Hartree–Fock procedure. In this way, we can use neutral or non-neutral atoms, once we have obtained information on the net charge in each individual atomic species from other computational programs, as done in this work.^61,72^ The MXAN procedure has also the possibility to fit transient spectra, i.e., the difference between the XANES spectra of a laser excited sample minus the unexcited one (LS state). This method reduces the influence of possible systematic errors in the experiment and calculations and at the same time strongly increases the sensitivity of the data to small changes.^61,72^

The fit procedure is performed in the energy space without using any Fourier transform, and the optimization in the space of the parameters is achieved minimizing the function

$$R_{sq}=n\;\frac{\sum_{i=1}^{m}w_{i}{\left[(y_{i}^{th}-y_{i}^{\;\text{exp}\;})\varepsilon_{i}^{-1}\right]}^{2}}{\sum_{i=1}^{m}w_{i}},$$

where n is the number of independent parameters, m is the number of data points, $y_{i}^{th}$ and $y_{i}^{\;\text{exp}}$ are the theoretical and experimental values of the absorption, respectively, $\epsilon_{i}$ is the estimated error in each point of the experimental data set, and $w_{i}$ is a statistical weight. In our case, $w_{i}=1$ and therefore, the square residual error function $R_{sq}$ becomes the standard $\chi^{2}$ function.

The latest version of MXAN also has the ability to optimize the two nonstructural parameters of the theory, which are the muffin-tin radii and the interstitial potential (see Ref. 61 for details). This is particularly important when comparing fits where the geometric structure has been derived from quantum mechanical calculations, as in this case, or from structures derived from molecular dynamics simulations. In this case, there is no further geometric adjustment of the derived structure as the fit is done only on the two nonstructural parameters. Therefore, this type of fit has always the same number of parameters.

## III. RESULTS AND DISCUSSION

In the following, we will present the results of the calculations and benchmark them against experimental observables for both the ground LS and the HS states.

### A. Molecular and electronic structure

**FIG. 1.**
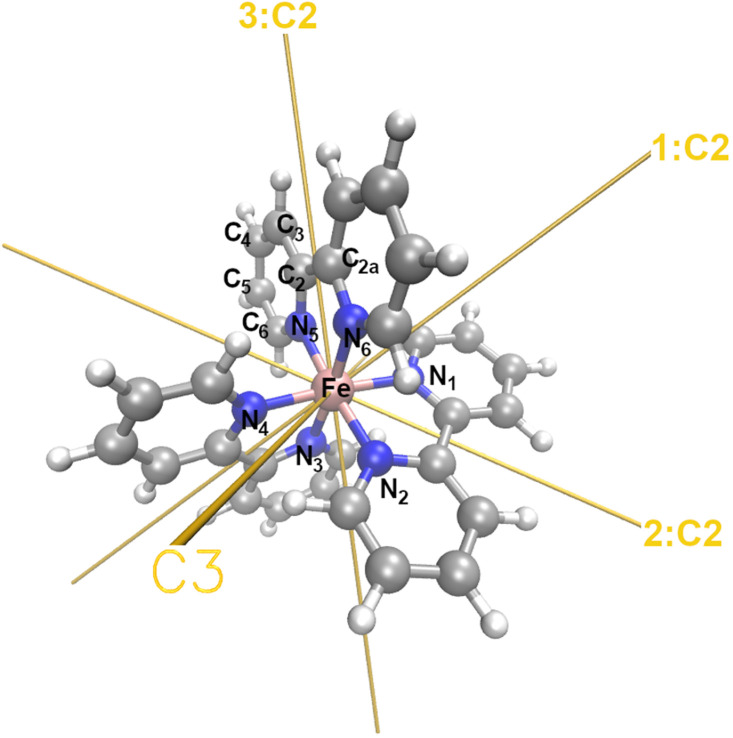
Atom labeling for definition of molecular geometry parameters of [Fe(bpy)_3_)]^2+^. Note the symmetry (C_3_ and C_2_) axis.

The PBE^*^/6–311+G^**^ optimized geometry of LS [Fe(bpy)_3_)]^2+^ in water dilute solution is shown in Fig. 1, along with the relevant atoms to define its molecular structure parameters. Details about distances and angles are given in Table I where they are compared with the values obtained by the C-PCM molecular geometry optimization and those from x-ray crystallography.^41^ The minimum energy optimization of the singlet spin state generates a D_3_ molecular geometry at all levels of computations, which was preserved throughout the optimization steps without imposing any geometrical constraints. As a consequence, all Fe–N distances, in plane (N5–Fe–N6, Fe–N5–C2, Fe–N5–C6) and out-of-plane (N3–Fe–N5–C2) angles are equal for all bipyridine ligands. These results agree with the QM/MM simulations of the last ten years,^39,73,74^ showing that a DFT approach in the free-molecule approximation, provides a fairly good agreement with the crystallographic structure (Table I), mostly evident on the Fe–N distance but overall, on the value of the Root-Mean-Square Deviation (RMSD) of 0.203 Å over all bond distances of the molecules (gas phase vs x-ray). Optimized D_3_ geometries of the isolated complex in the LS state (^1^A_1_) reported Fe-N distances of 1.997 Å using the hybrid BHLYP functional;^74^ with the same hybrid functional, the same authors reported an average value for the Fe-N distance—computed considering a nanoseconds timescale Car-Parrinello Molecular Dynamics (CPMD) in water dilute solution in a cubic box—of 1.982 Å with a standard deviation (*σ*) of 0.056 Å. Our simulations by using an all-electron approach report a Fe–N value of 1.966 Å in aqueous environment and of 1.971 Å in gas phase (see Table I). Moreover, the C2-C2a (see Fig. 1) distances of almost 1.466 Å also result in agreement with the statistical distribution extracted from CPMD-based sampling (average value = 1.466 Å, *σ* = 0.030 Å). In addition, the distribution of the N_(1,3,5)_-Fe-N_(2,4,6)_ angle being centered at 81.5° with a *σ* = 2.1° well agrees with the detected value of 81.7° at PBE^*^/6–311+G^**^ level of theory for the [Fe(bpy)_3_)]^2+^complex.

**TABLE I.** PBE^*^/6–311+G^**^ gas phase (C-PCM/water in parenthesis) structural parameters of [Fe(bpy)_3_)]^2+^ low-spin (LS) ground state and high-spin (HS) excited state. Distances in Å and angles in degrees. Crystal is the crystallographic structural data from Ref. 41. XANES^5^ is the structural determination coming from fitting the transient experimental XANES data using the MXAN procedure and assuming a D_3_ symmetry.

	Low-spin ground state		High-spin excited state	
	Calculated	Crystal^41^	Calculated	XANES^5^
Fe–N1	1.971 (1.966)	1.967	2.181 (2.173)	2.206
Fe–N2	1.971 (1.966)	1.967	2.193 (2.185)	2.206
Fe–N3	1.971 (1.966)	1.967	2.166 (2.162)	2.206
Fe–N4	1.971 (1.966)	1.967	2.169 (2.164)	2.206
Fe–N5	1.971 (1.966)	1.967	2.183 (2.174)	2.206
Fe–N6	1.971 (1.966)	1.967	2.192 (2.186)	2.206
N–C2	1.371 (1.371)	1.359	1.362–1.364 (1.360–1.363)	1.359
N–C6	1.355 (1.354)	1.338	1.350–1.352 (1.348–1.351)	1.338
C2–C2a	1.466 (1.465)	1.471	1.479–1.484 (1.477–1.483)	1.378
C2–C3	1.402 (1.401)	1.377	1.404–1.405 (1.403–1.404)	1.378
C3–C4	1.395 (1.394)	1.374	1.394–1.396 (1.393–1.395)	1.374
C4–C5	1.399 (1.398)	1.380	1.398–1.399 (1.398–1.399)	1.380
C5–C6	1.394 (1.393)	1.358	1.393–1.395 (1.393–1.395)	1.357
N5–Fe–N6	81.5 (81.7)	81.8	75.5 (75.8)	89.7
N3–Fe–N4	81.5 (81.7)	81.8	75.6 (75.6)	89.7
N1–Fe–N2	81.5 (81.7)	81.8	75.9 (75.6)	89.7
N1–Fe–N4	175.2 (175.5)	174.6	167.2 (167.6)	174.6
N2–Fe–N5	175.2 (175.5)	174.6	171.2 (171.2)	174.6
N3–Fe–N6	175.2 (175.5)	174.6	167.8 (167.9)	174.6
Fe–N5–C2	115.4 (115.3)	115.0	116.4 (116.5)	115.0
Fe–N5–C6	126.4 (126.4)	127.6	125.2 (124.7)	127.6
N2–Fe–N5–C2	−44.9 (44.9)	−42.9	−47.6 (−45.6)	−42.9
N3–Fe–N6–C2a	−44.9 (44.9)	−42.9	−50.4 (−49.2)	−46.7
N4–Fe–N1–C2b	−44.9 (44.9)	−42.9	−46.5 (−43.9)	−46.7

**FIG. 2.**
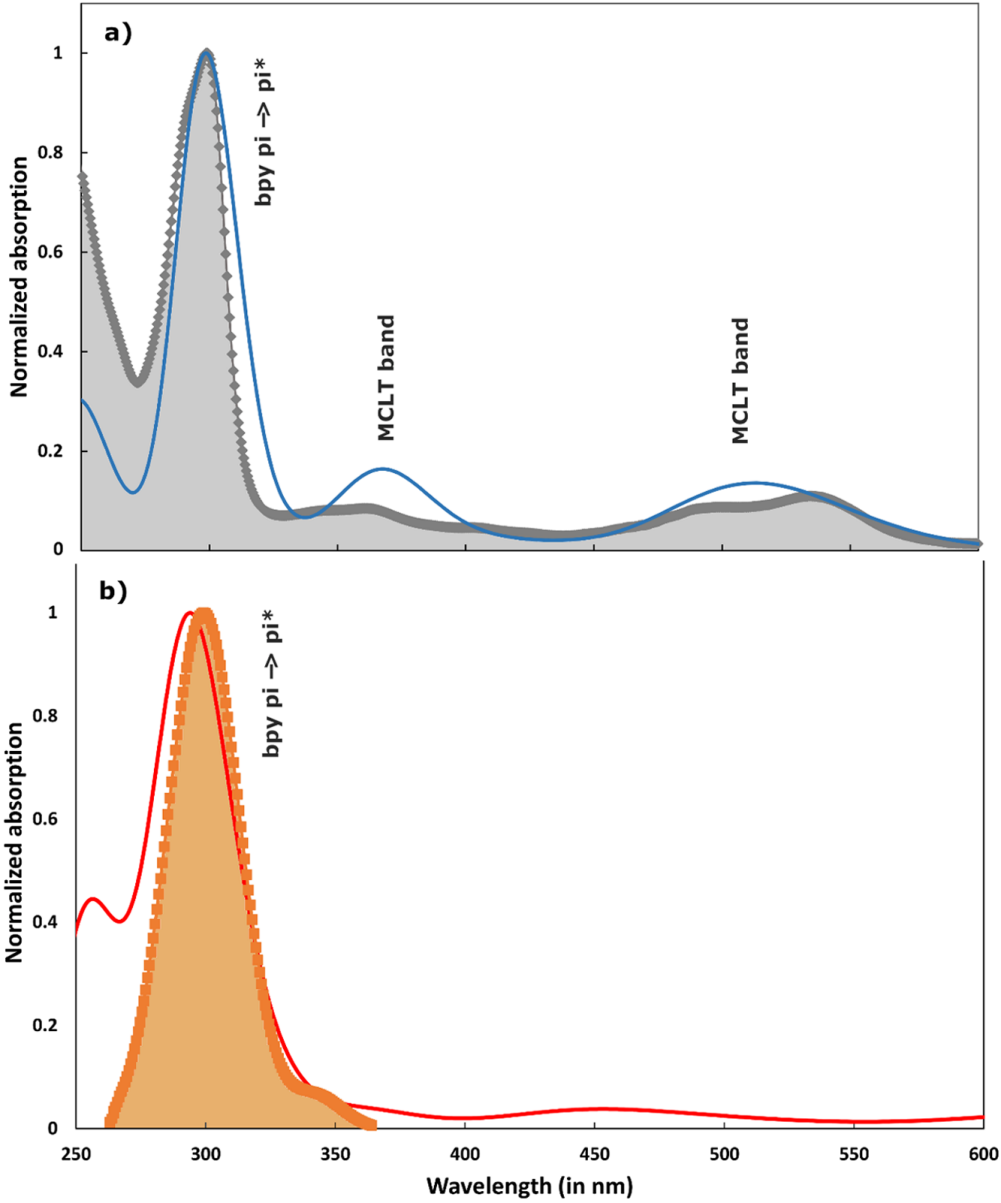
(a) UV/Vis computed absorption spectra for the LS state (blue line), estimated at TD-DFT PBE^*^/6–311+G^**^ level of theory of [Fe(bpy)_3_)]^2+^ in trichloromethane solution via C-PCM model, and compared to the experimental spectrum in trichloromethane solution at room-temperature (Gray diamonds). (b) Same for the computed HS state spectrum (red line: C_1_ 57.9% and C_2_ 42.1% at 298 K) and the experimental spectrum (orange squares) retrieved from pump-probe data.^56^

**FIG. 3.**
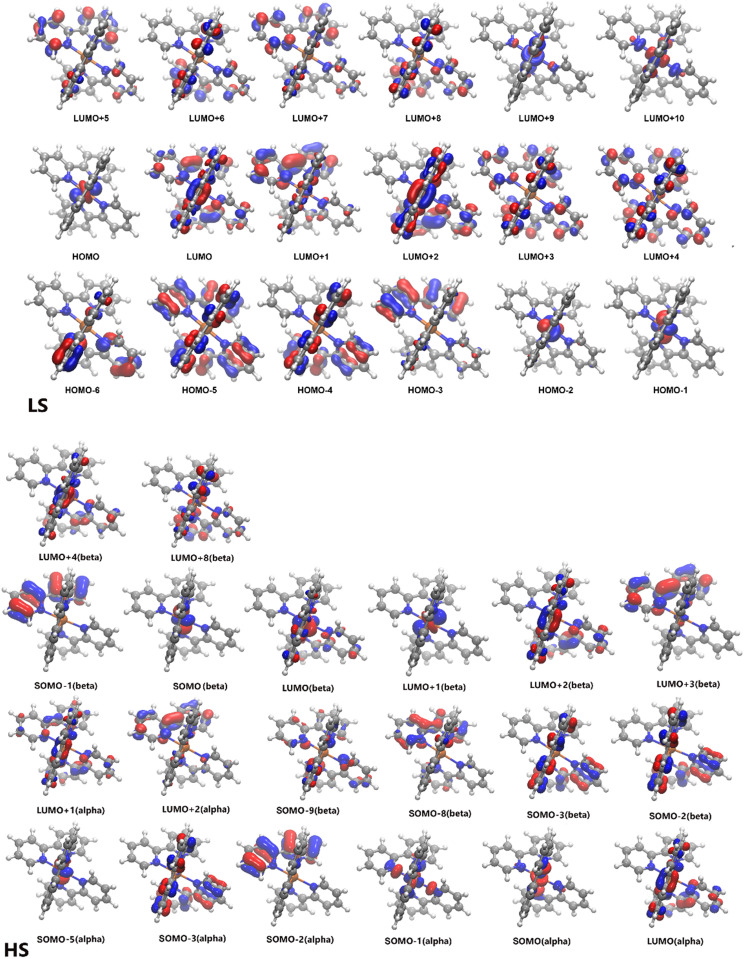
Molecular orbitals involved in the absorption spectrum (see Fig. 2) for the LS and HS state of the [Fe(bpy)_3_)]^2+^complex as estimated at PBE^*^/6–311+G^**^ level of theory in conjunction with C-PCM model (TCM dilute solution). Blue color reflects the positive (+) counterpart of the corresponding eigenvector; an isodensity surface value of 0.05 e·Å^−3^ was used. LS: the corresponding eigenvalues are (in eV): −13.59, −12.29, −12.21, −12,21, −10.94, 10.94, −10.78 (HOMO), −8.17 (LUMO), −8.15, −8.15, −7.53, −7.21, −7.21, −7.21, −7.05, −7.05, −6.54, −6.54. HS(*α*): eigenvalues (in eV): −12.31, −12.12, −12.05, −11.19, −11,02 (SOMO), −8.03, −7.99, 7.92; HS(*β*): eigenvalues (in eV): −13.43, −13.40, −12.14, −12.12, −12.05(SOMO), −10.15, −8.50, −8.06, −7.93, −7.89, −7.65, −6.89. With the term SOMO, we identified the highest molecular energy singularly occupied with alpha or beta spin multiplicity, respectively.

Using this geometry, we compute the ground state TD-DFT optical absorption spectrum at PBE^*^/6–311+G^**^ level, which is compared in Fig. 2(a) with the experimental absorption spectrum for [Fe(bpy)_3_)]^2+^ system at room temperature^8^ and with the one computed with C-PCM method in trichloromethane (TCM) solvent (see Fig. S1 of the supplementary material for comparison with DCM and TCM solvents) after geometry re-optimization. The molecular orbitals involved in the vertical excitation processes for LS multiplicity state are displayed in Fig. 3. Our computed spectra are in line with the experimental data. In fact, the bands at ∼350 and ∼500 nm in the experimental spectrum are at 364 nm (oscillator strength, f = 0.062) and 505 nm (f = 0.066), respectively, in the computed one. Moreover, as expected, these excitations are accompanied by a significant charge transfer (CT) character. The former mainly involves a linear combination of the following single excitations at electronic level: HOMO-2→LUMO + 4, HOMO→LUMO + 7, HOMO-1→LUMO + 4 and HOMO→LUMO + 8, while the latter (known for its ^1^MLCT character) shows a multiple excitation character involving—with different weights—the HOMO-2, HOMO-1, LUMO and LUMO + 1 orbitals. Looking at the molecular orbitals in Fig. 3 (upper panel), it is obvious that the unoccupied molecular orbitals reflect a pure valence π^*^ character. Last, the strongest absorption band experimentally detected around 300 nm in a TCM solution with a shoulder at shorter wavelengths is theoretically estimated as a single band showing a maximum at ∼291 nm. A deeper analysis actually reveals that, this band is mainly composed of absorption features at 288 nm (f = 0.59) and 294 nm (f = 0.17). The former signal is essentially composed of HOMO-5→LUMO, HOMO-4→LUMO + 2 and HOMO-3→LUMO + 1 excitations, while the latter of HOMO-5→LUMO + 2 and HOMO-5→LUMO + 1 single character excitations. In summary, we get a remarkable agreement between experimental and computed spectrum in the 200–700 nm region, not only in terms of band energies but also of relative intensities.

**FIG. 4.**
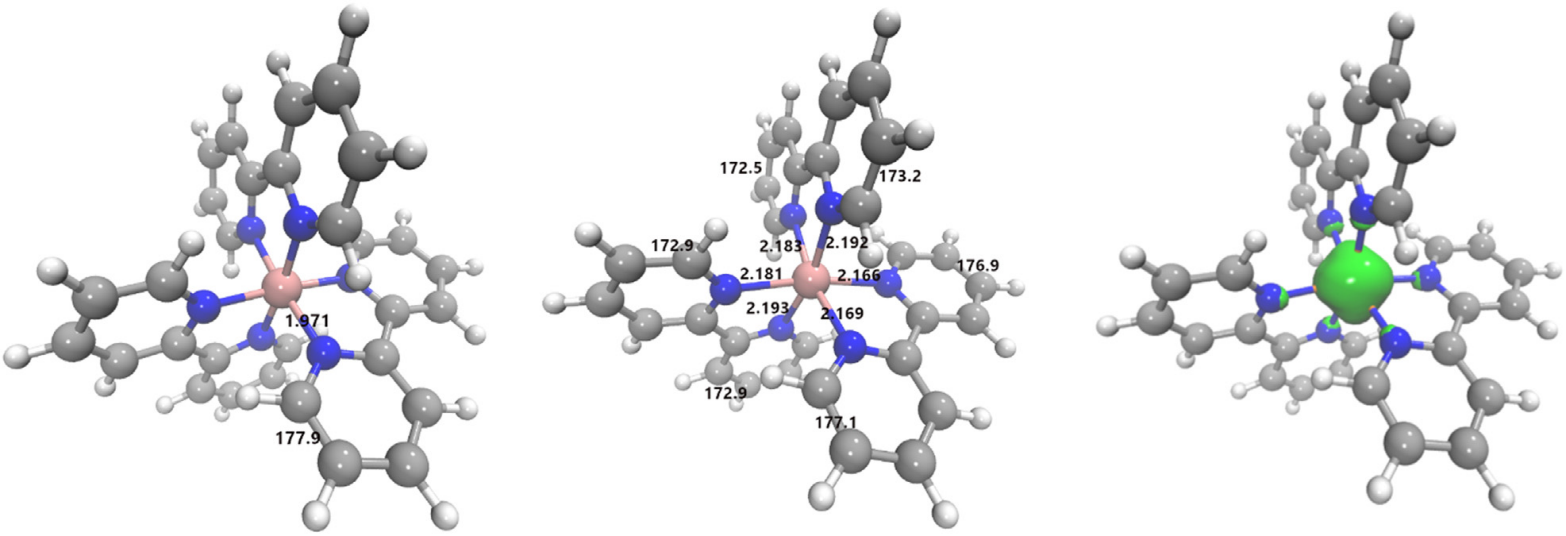
Key structural parameters of [Fe(bpy)_3_)]^2+^ (Fe–N bond distances in Å and pyridine out-of-plane angles in degrees) optimized at PBE^*^/6–311+G^**^ level of calculation in gas phase. Left: LS. Center: HS. In the right panel, we also display for the HS state the Electron Spin Density by Difference (ESD-D) surface in green color with an isovalue of 0.01 e Å^−3^.

Having benchmarked our computational approach against the LS molecular structure and its UV-Visible absorption spectrum, we calculate the HS state most stable molecular structure using the same PBE^*^/6–311+G^**^ model and computational procedure. From the initial steps of the optimization, the geometry of this state quickly diverges from the D_3_ symmetry and ends up in a non-symmetric molecular structure after releasing the major structural parameters around the central Fe^2+^ ion. The resulting optimized geometry parameters are also given in Table I, and Fig. 4 shows the differences between the HS ${[}^{5}A(t_{2g}^{4}e_{g}^{2}-\text{like})]$ and LS ${[}^{1}A_{1}(t_{2g}^{6}e_{g}^{0})]$ structures, referred here (and in the following discussion) to the PBE^*^/6–311+G^**^ level of calculation in the gas phase. In the same figure, we report—for the HS state of the [Fe(bpy)_3_)]^2+^complex—the Electronic Spin Density by Difference (ESD-D) surface which essentially allow us to derive that the two unpaired electrons characterizing the quintet multiplicity result localized over the central iron (II) atom. For completeness, as for the LS state, the computational pipeline for the HS state has been carried out also in the solvent using C-PCM and for a quick comparison the most relevant (in water solvent) optimized structural parameters are also reported in Table I. The resulting HS structure shows three sets of different Fe–N bond distances with mean values of 2.182, 2.192, and 2.167 Å and slightly distorted in-plane and out-of-plane angles leading to a C_1_ symmetry. In addition, we also considered the HS state in a C_2_ symmetric sub-space. Such a simulation allows us to identify an optimized structure for such a species featuring interatomic distances resembling those already appreciated by Sousa and coworkers in Ref. 21 (for instance, the Fe–N_1_, Fe–N_3_ and Fe–N_5_ previously estimated in the gas-phase at 2.190, 2.210, and 2.198 Å at PBE0/def2-TZVP level are found lying at 2.171, 2.189, and 2.183 Å in DCM). Furthermore, the C_2_ symmetric structure is found at a higher potential energy by 0.19 kcal/mol when compared with the predicted asymmetric counterpart. It is therefore evident that thermal fluctuations at finite-temperature conditions will lead to a population ratio suggesting the coexistence of both symmetric (C_2_) and asymmetric HS conformations. Moreover, such an energy gap is found to be in line with the lowest energy bending normal frequencies of the bpy ligands in the HS state multiplicity.

The difference between LS and HS structures can be appreciated by focusing on the most relevant parameters characterizing the iron-bipyridine interaction in Table I, namely, the Fe–N distance and the in-plane and N–Fe–N–C dihedral angle. The latter expresses the extent to which the iron atom is in the plane defined by the aromatic ligands. Evidently, for the LS ground state only one value is reported common to all ligands, because of the D_3_ symmetry. In the HS state, the Fe–N interactions are weakened (their distances increase by about 0.2 Å) as a result of the antibonding character of the e_g_ [SOMO-1(*α*), SOMO(*α*)] orbitals depicted in Fig. 3, lower panel. In Table I, we see that the Fe–N distances in ideal gas-phase split into three sets, namely, 2.181–2.183, 2.192–2.193, and 2.166–2.169 Å (with an average distance of 2.181 Å and a standard deviation of 0.011 Å) and correspondingly the bipyridine in plane N-Fe-N angle of 175.2° in D_3_ symmetry of the GS splits into three values: 167.2°, 167.8°, and 171.2°, conferring a low symmetry final structure to the HS state. The complex appears as an axially elongated octahedral complex in which the equatorial bonds are, however, equivalent in pairs, both of them (the shorter ones) being located on the same ligand and the other two (the longer ones) on the remaining two ligands. In the water solution, modeled via C-PCM approach, the average value of the Fe–N interatomic distance is equal to 2.174, with a standard deviation of 0.010 Å (see data collected in Table I), i.e., shorter. The observed distortions agree with data available in the literature using pseudopotentials which imply in water solutions changes of 0.01–0.02 Å in the Fe−N bond lengths and of ca. 4° in the N−Fe−N^*^ bending angle for complex, upon a LS → HS change.^74^ It is also interesting to observe that a connection exists between shorter (and thus stronger) Fe–N bonds and the Fe–N–C–C dihedral angles close to 180°. The nitrogen lone pairs coordinating the central iron atom lie in the planes of the aromatic rings and the dihedral planarity permits to maximize the superposition with the receiving orbitals of the Fe atom. The same considerations obviously hold for a *π*-back bonding interaction with the π^*^ bipyridine orbitals.

In Ref. 56, the absorption spectrum of the HS state was extracted for the 260–360 nm region. The C-PCM (in TCM) UV-Vis absorption spectrum of the HS state simulated via TD-DFT, is compared to the experimental one in Fig. 2(b) (see Fig. S2 for a comparison with the gas phase computation). It is estimated for a mixture of C_1_(57.9%) +C_2_(42.1%) structures, using a population ratio derived from the energy difference of 0.19 kcal/mol at 298 K; moreover, given the slight difference observed between the two structures, the corresponding vertical UV/Vis absorptions are indeed very similar (differences are confined within a few nanometers). The simulated HS spectrum shows a dominant ^5^*π*→π^*^ ligand charge (LC) band in the 300 nm region and confirms the absence of intense absorption bands in the >350 nm region in agreement with the experimental spectrum, but also with that of other Fe(II) SCO complexes, whose HS states can be stabilized at low temperatures.^2^ Such a prominent peak mainly arises from three quintet electronic transitions with oscillator strengths of 0.12, 0.19 e 0.22 au. with the following composition: SOMO-9(*β*)→LUMO(*β*), SOMO-8(*β*)→LUMO(*β*), SOMO-2(*β*)→LUMO + 4(*β*), SOMO-2(*α*)→LUMO + 2(*α*), SOMO-1(*β*)→LUMO + 3(*β*), see the molecular orbitals depicted in Fig. 3, lower panel. We also note that the theory predicts a slightly blue-shifted LC band compared to the experiment.^56^ The remaining low-intensity bands (with oscillator strengths of ∼0.01 a.u.) at longer wavelengths in the 350–600 nm range fall at 362 and 445 nm; these two signals resulting substantially populated by SOMO(*β*)→LUMO + 8(*β*), SOMO(*β*)→LUMO + 4(*β*), SOMO-5(*α*)→LUMO + 2(*α*), and SOMO(*α*)→LUMO + 2(*α*) may be then assigned as ^5^MLCT transitions.^2,75^ In addition, we would like to highlight that our TD-DFT computations estimate two low-lying quintet excited state at 5705 and 4787 nm resulting from SOMO(*β*)→LUMO(*β*) and SOMO(*β*)→LUMO + 1(*β*) permutation, respectively (see Fig. 3, lower panel). Such a character in the excitation patterns qualitative agrees with the analysis recently reported by Lee and co-workers with a symmetric counterpart (i.e., ^5^T_2_) of the [Fe(bpy)_3_)]^2+^ system at DFT level in acetonitrile solution (see Table S11 in Ref. 76).

To summarize this part, both the LS and HS optical absorption spectra are well reproduced by the present computation. The above discussion deals with stabilized structures. However, strictly speaking ensemble averaging would be needed given the floppy nature of the molecule.^22,74^ This is especially important for the torsional angles of the Fe(II) complex in solution, for which the distribution of conformations is expected to be broad, as experimentally confirmed through time-resolved UV anisotropy and CD measurements.^56^ From a computational point of view, structural averaging the geometries of LS/HS conformers is a particularly involved task, which is beyond the scope of the present paper. Rather, the stabilized structures obtained above are considered the most probable ones for the respective states, although a thorough consideration of the distribution of conformations would be needed in the future.

### B. X-ray absorption spectra

This section is dedicated to the comparison between the LS ground state and HS excited state XANES experimental data with the calculations obtained using the stabilized structures obtained in Sec. III A. This is achieved through the MXAN procedure, where the fits are carried out only on nonstructural parameters, as specified in Sec. II B.

**FIG. 5.**
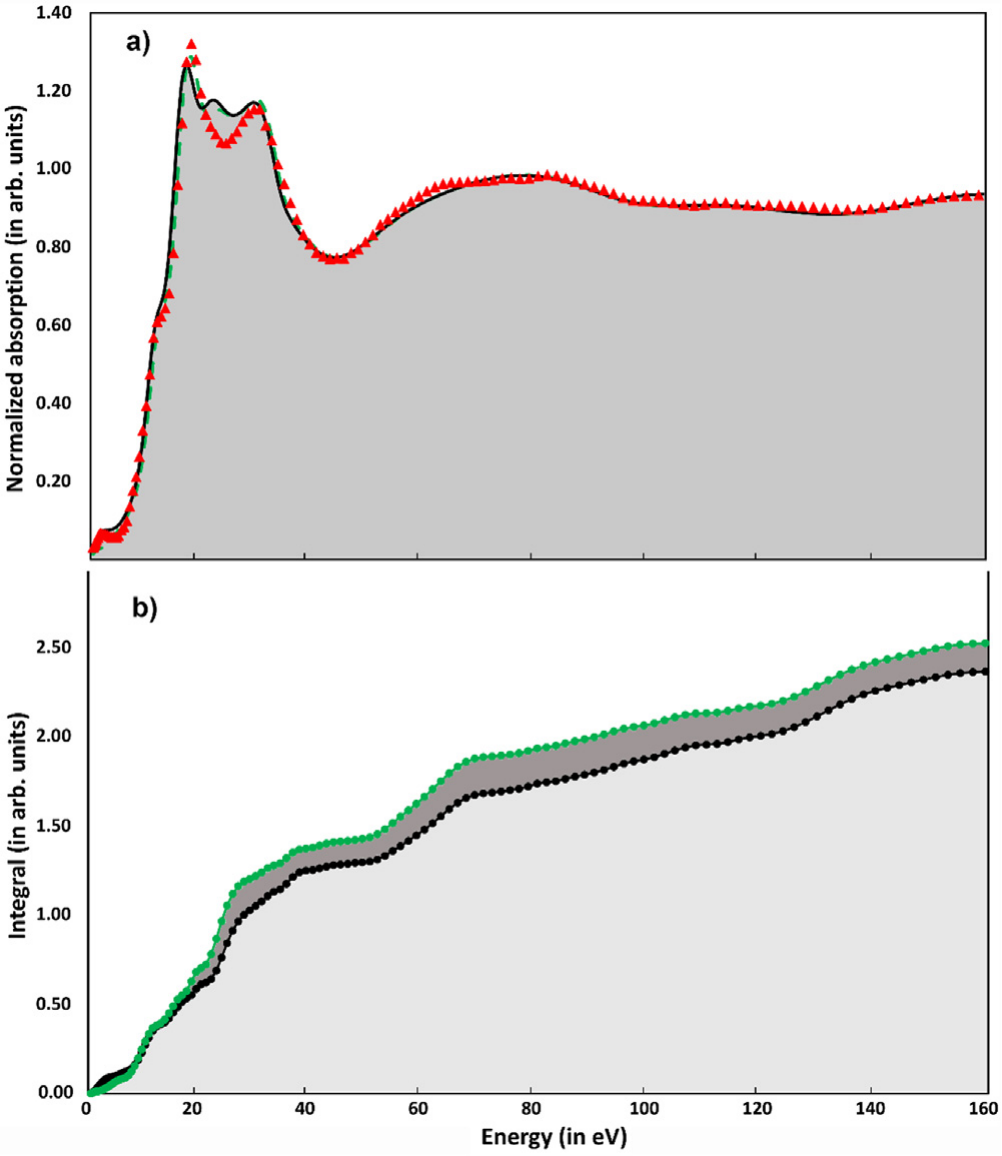
(a) LS experimental XANES spectrum of [Fe(bpy)_3_)]^2+^, plotted as excess energy above the Fermi energy (red triangles), and two calculations at the best fit condition for PBE^*^ (black trace) and PBE^*^ with RESP charges (green dashed line) geometries; (b) integrated area of the fit error function f_e_(E) in normalized area units. Again, the black and the green profile refer to PBE^*^ computations with and without RESP charges, respectively.

We first benchmark our theoretical results on the LS XANES spectrum. The comparison between the experimental data (red dots) and the PBE^*^ derived geometric structures at the best-fit conditions (see Sec. II B) is shown in Fig. 5. The fit obtained using the PBE^*^ geometry yields R_sq_ = 3.18. The agreement between the experimental data and the theoretical calculation is quite good over the whole energy range, with a small discrepancy due to the presence of an extra peak in the energy region about 10 eV after the edge. This good agreement is consistent with the PBE^*^ Fe–N value being close to that of the crystallographic data,^41^ the fit of the XANES spectrum done by moving the geometric structure,^5^ and the fit of the EXAFS spectrum,^42^ all of which yielded identical values (2 ± 0.02 Å).

To go deeper into the comparison between experimental data and theoretical calculations, we also explore the possibility of using non-neutral atoms in the MXAN calculations. In this case, MXAN has the possibility to associate a net charge value in every single atomic species present in the cluster of atoms used for the calculation. In this way, we can mimic the presence of bonds between the atomic species in the potential used to calculate the absorption spectra. Each atom in the PBE^*^ geometry has its own RESP charge reported in Table S1. The calculation with RESP charges at the best fit condition is reported in Fig. 5 as a green dashed line. The use of the RESP charges reduces the error R_sq_ from 3.18 to 2.42. The improvement is mainly confined to the first 30 eV from the edge where details of the potential can be important. This is also confirmed by the behavior of the integral (as a function of energy) of $f_{e}\left(E\right)$ defined as

$${\;f}_{e}\left(E\right)=\sqrt{(y_{th}\left(E\right)-y_{\;\text{exp}\;}(E){\;)}^{2}},$$

where $y_{th}\left(E\right)$ and $y_{\;\text{exp}\;}(E)$ are the values of the fit and the experimental data at different energies. This function is useful to discriminate between various fits in all cases where the difference in the residual R_sq_ function is small.^77^ For PBE^*^ geometry with RESP charges it is always smaller than the one corresponding to the PBE^*^ case without charges in the whole energy range [see Fig. 5(b)]. The fact that using non-neutral atoms reduces the value of the error R_sq_ is a further confirmation of the quality of the PBE^*^ geometry as it indicates a better charge distribution. It should be noted that this way of associating each atom with the value of its net charge does not increase the number of parameters in the fit procedure, which is always 2 (interstitial potential and muffin-tin radii) in all fits presented in this work.

We now turn to the case of the HS state. In previous analyses,^5,42^ the fit of the differential XANES spectrum (excited minus unexcited XANES spectrum at 80 ps time delay) was carried out assuming a D_3_ symmetry also in the HS state. The resulting Fe–N bond increase in the HS state was found to be ∼0.2 Å. However, we now see through the PBE^*^ calculations that a departure from the LS D_3_ symmetry occurs in the HS state, with a non-negligible distortion due to inequivalent Fe–N bond elongations (Table I), although the average Fe–N distance remains ∼2.18 Å, in agreement with Refs. 5 and 42.

**FIG. 6.**
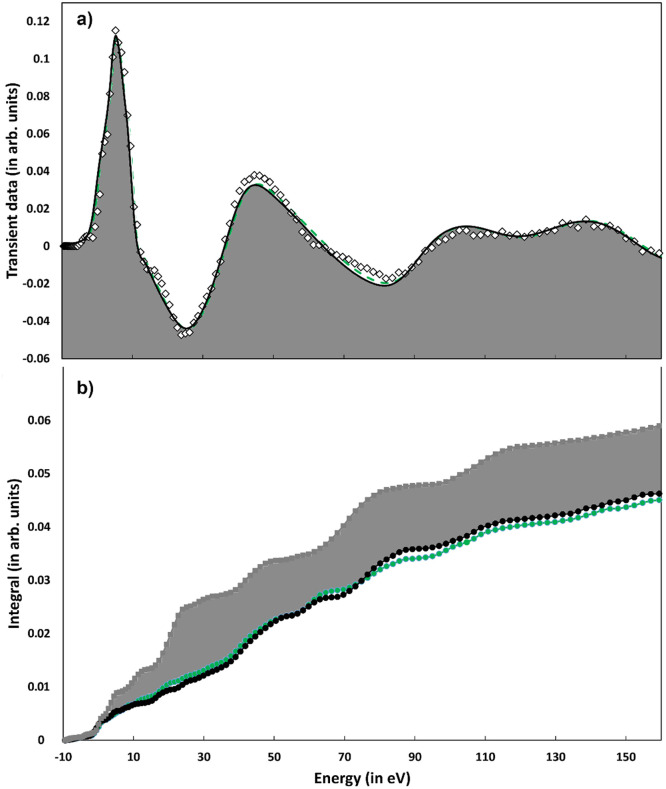
(a) Comparison between the experimental differential transient data (white diamond) and MXAN fitting with (green dashed line) and without (black line) RESP charges for the PBE^*^ HS state geometry. (b) HS excited state integrated area of the fit error function f_e_(E) in normalized area units. Gray profile: fit imposing a D_3_ symmetry of Ref. 5 black profile: PBE^*^ geometry fit, no RESP; green profile: PBE^*^ geometry fit with RESP.

In order to test the results of the PBE^*^ structural determination for the HS state, we simulate the differential transient data using the optimized HS geometry following the same procedure used for the LS state that is, at fixed molecular geometry without any further molecular structure refinement to improve the overall fit. Figure 6(a) compares the transient experimental data^64^ (black diamonds) and two calculations made with the PBE^*^ geometry and with (green dashed line) and without (black solid line) RESP charges. As for the ground state, the use of RESP charges slightly improves the quality of the fit (the error function R_sq_ decreases from 1.38 to 1.26). In both calculations, the value of R_sq_ is much lower than 2.97, which is the one found with the global fit (structural in a D_3_ symmetry and nonstructural parameters) procedure used in Ref. 5. The value of 2.97 is obtained using the more recent experimental transient data with a higher signal to noise ratio.^64^ In other words, the PBE^*^ structure reproduces the transient experimental data better than the ones in Ref. 5. To further highlight the improved agreement (with respect to the fit done imposing the D_3_ symmetry^5^) between the experimental data and the calculations, using PBE^*^ nonsymmetrical geometry with and without charges, Fig. 6(b) shows the comparison between the integral of the *f_e_*(*E*) function for the three fits. The calculations releasing the D_3_ symmetry constraint show a significant improvement and among these the one using the PBE^*^ geometry with RESP charges produces a lower integral in a wide energy range, confirming—as already reported in the literature using pseudopotentials—the preference for the distorted geometry and an asymmetric charge distribution.

### C. Transient ultraviolet circular Dichroism spectra

**FIG. 7.**
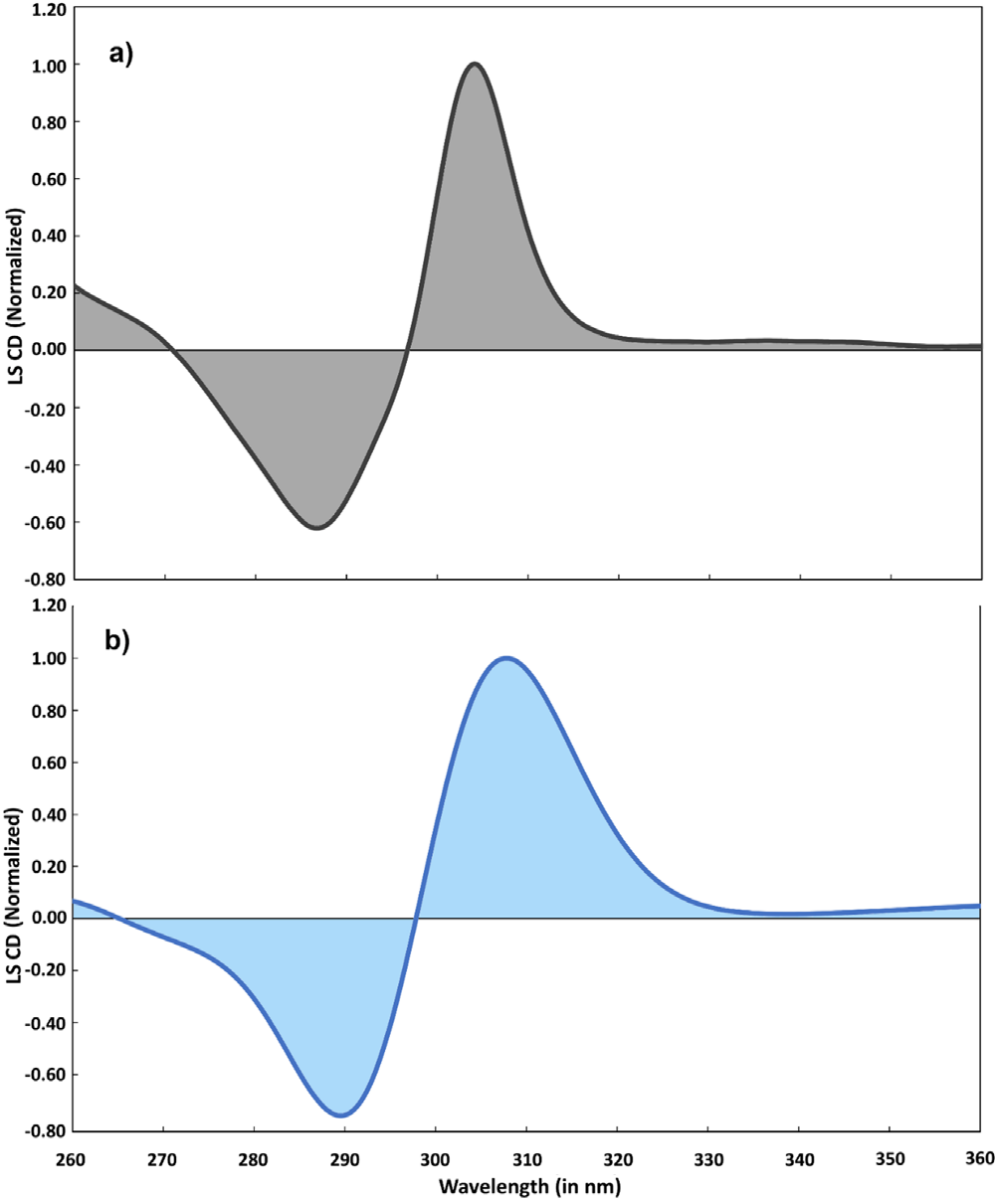
(a) Experimental circular dichroism (CD) spectrum^56^ of the LS state of [Fe(bpy)_3_)]^2+^ at room-temperature in trichloromethane dilute solution, in the 260–360 nm range; (b) simulated CD spectrum—at TD-DFT PBE^*^/6–311+G^**^ level of theory in conjunction with C-PCM model in trichloromethane applying a FWHM broadening of 0.4 eV (see the text).

In order to complement the above analysis of the XANES, we also carried out simulations of the recently reported steady-state and time-resolved deep-UV CD spectra.^56^ In this work, the molecules were the very similar iron(II)tris(4,4′-dimethyl-2,2′-bipyridine), associated for stereocontrol with the enantiopure Δ or Λ-enantiomer of tris(3,4,5,6-tetrachlorobenzene-1,2-diolato-*κ*2O1,O2)phosphorus (V) (P(O_2_C_6_Cl_4_)^3–^ or TRISPHAT) anions.^78^ As seen in Fig. 2, the LS absorption is dominated by the intense LC band around 300 nm. This band is sensitive to the chiral conformation of the molecule, and the CD signal stems from the coupling of the three dipoles that are oriented along the long axis of the bpy ligands.^56,79^ Figure 7(a) shows the experimental ground state CD spectrum (in TCM solvent) in the region of the LC band, and Fig. 7(b) shows the simulated one, using the same structural parameters and methods as those used for the UV-Visible absorption and the XANES spectra, optimized for the TCM solvent. Figure S3 shows the simulated LS CD spectra for both water, DCM and TCM solvents. The water spectrum is slightly blue shifted with respect to the other two solvents, reflecting the same trends as in the linear absorption spectrum (Fig. S2). The agreement in Fig. 7 is remarkable, bearing in mind that the deviations that occur have the same origin as those in the UV-Visible absorption spectrum (Fig. 2). In particular, the dissymmetry between the red positive and blue negative lobes of the signal is well reproduced.

**FIG. 8.**
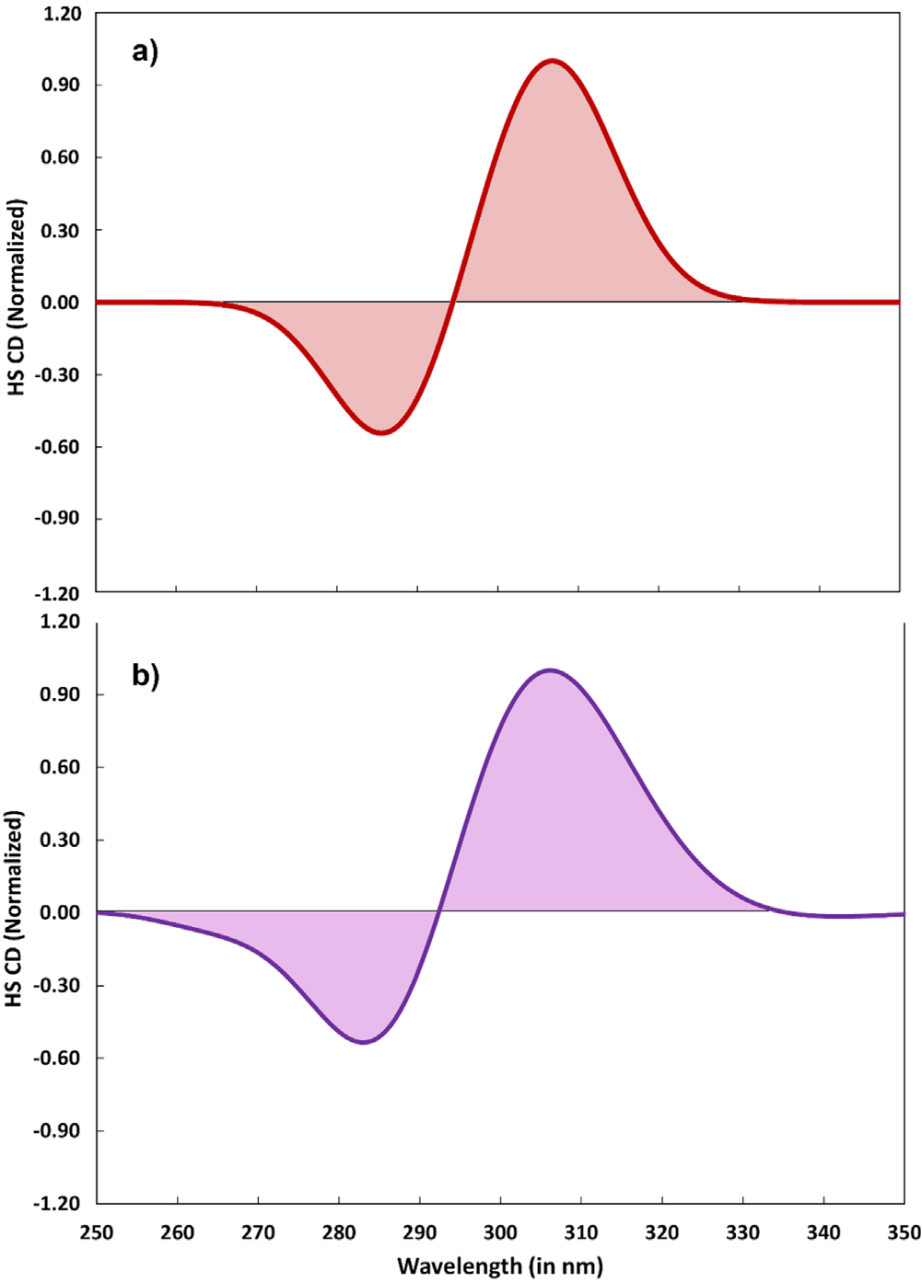
(a) Experimental CD spectra^56^ at room-temperature in dichloromethane dilute solution for the HS form of the of [Fe(bpy)_3_)]^2+^ compound in the range between 250 and 350 nm; (b) Simulated CD spectrum—at TD-DFT PBE^*^/6-311+G^**^ level of theory in conjunction with C-PCM model—of the HS state (C_1_ 57.9% and C_2_ 42.1% at 298 K) in dichloromethane, applying a FWHM broadening of 0.4 eV (see the text).

We now turn to the case of the HS state. Figure 8(a) shows the experimental CD spectrum of the HS state retrieved from the pump-probe spectrum at 4 ps^56^ in DCM and Fig. 8(b) shows the calculated one in the same solvent, using the same electronic and structural parameters that were used to simulate the UV-Visible absorption (C_1_+C_2_) and the XANES spectra. Figure S3 compares the simulated HS CD spectra in various solvents. As for the LS state, we also observed a slight blue shift of the water spectrum with respect to the other two solvents. Figure S4 presents the same comparison as in Fig. 8 but using TCM C-PCM modeled calculations. Concerning Figs. 8 and S4, one notes that peak positions of the HS state are well reproduced by the simulation, as are the relative intensities of the two lobes. There are also some deviations, in particular in the experimental spectrum a shoulder appears on the blue positive lobe just above 290 nm, which is not reproduced in the simulations, as was the case with the UV-Visible absorption spectrum (Fig. 2). It may be due to an additional state, not accounted for in our calculations, or to a vibronic band of the LC transition. In summary, our simulated CD spectra agree very well with the experimental ones both in the case of the LS state and the HS state. Most importantly, this agreement is achieved using the same electronic and structural parameters as used for the UV-Visible and the XANES spectra. Therefore, the electronic and structural characteristics of the system in the HS state are supported by two very different types of observables.

### D. Vibrational spectroscopy

One of the goals in the study of the vibrational spectrum of the two electronic states of the [Fe(bpy)_3_]^2+^ complex is to characterize the stationary point(s) optimized at the PBE^*^/6–311+G^**^ level as local minimum(a) of the potential energy surfaces (PES). Ultrafast transient absorption spectroscopy have revealed a number of (low-frequency) vibrational wave packets generated in the forward SCO process,^8,15^ while transient IR spectroscopy has identified a number of high frequency modes in the 1200–1600 cm^−1^ region. In the latter, the experimentally derived modes were compared to DFT calculated ones for the ground state, the intermediate ^3^T state and the ^5^T state.

We present a complete analysis (at the highest level made possible by today's computing resources—DFT with a very extensive basis set) of the vibrational spectrum starting from the calculation of the normal modes of vibration with the study of the Potential Energy Distribution (PED) of both the LS and HS state. In fact, it is thanks to the theoretical study of the vibrational normal modes and the differences between the LS and HS states that we can hypothesize which variations in the internal coordinates between the two states are the most significant in breaking the D_3_ symmetry in the HS states. It should also be noted that the use of vibrational normal modes has been a key factor in the interpretation of the time-dependent XANES spectra of Fe(II)-SCO complexes obtained at x-ray Free Electron Lasers.^17^ Last but not least, in our opinion this analysis could hopefully pave the way to further theoretical and experimental studies aimed at assessing the key structural features of the strongly coupled vibronic states emerging in the transition from the LS to the HS state in [Fe(bpy)_3_]^2+^.

Of the 177 fundamental vibrational modes of the LS D_3_ form, 30 are A_1_ Raman-active, 29A_2_ IR-active and 59 E IR- and Raman-active ones.^37^ The experimental FTIR and Raman mode frequencies of LS [Fe(bpy)_3_]^2+^ were compiled in Ref. 37, based on references therein. The low- to medium-frequency modes here calculated at PBE^*^/6–311+G^**^ level in the gas phase are presented in Table II and compared with the experimental mode frequencies of the LS state. A more detailed PED analysis of the theoretical vibrational spectra of the ligand and of the two electronic states of the complex is provided in Tables S3(a), S4(a), and S5. A further basis for the vibrational assignment of the infrared spectra was provided from the comparison of the theoretical spectra of the complexes with that of the bpy ligand (Table S2). The high frequency (especially above 500 cm^−1^) vibrational modes are due to the ligand.^46^ The discussion hereafter is restricted to the main differences between the LS and the HS states detected in the calculations, after benchmarking the latter against the low frequency modes observed in the HS state by ultrafast spectroscopy.^8,15^ Figure S5 shows the computed IR spectra of LS and HS states. In the following, we discuss separately the low frequency and high frequency regions of the vibrational spectrum.

**TABLE II.** Experimental, calculated frequencies (PBE^*^/6–311+G^**^ level in the gas phase; in cm^−1^) and LS P.E.D. analysis with assignments of the vibrational modes of [Fe(bpy)_3_]^2+^ in the low-spin (LS) and high-spin (HS) state.

	Calc. LS	Exp. LS^30^	PED analysis of LS state^a,b^	Calc. HS	exp. HS^15^
A_2_	497	470	13% *π*(NNNFe) + 12% *β*(CCC) + 12% *ρ*(CCCC) + 9% *β*(CNC) + 9% *π*(CCFeN) + 7% *ρ*(CCNFe) + 6% *β*(NCC)		
E	496	470	26% *ρ*(CCCC) + 17% *π*(CCFeN) + 9% *β*(NFeN) + 7% *π*(CCNC) + 5% *β*(CCC) + 5% *β*(NCC) + 5% *ρ*(CCNFe)		
E	470	470	22% *β*(NCC) + 20% *β*(CCC) + 9% *π*(CCFeN) + 8% *ρ*(CCCC) + 6% *β*(CNC)		
A_1_	455	470	25% *ρ*(HCCC) + 16% *π*(CCFeN) + 15% *ρ*(CCCC) + 9% *ρ*(CCCN) + 8% *β*(NFeN) + 8% *ρ*(CCNFe) + 5% *π*(CCNC)		
A_2_	455	470	16% *β*(CCC) + 11% *β*(NCC) + 9% *ρ*(CCNFe) + 9% *π*(FeCCN) + 7% *ν*(NC) + 7% *ρ*(CCCC) + 6% *ν*(FeN) + 6% *π*(CCFeN)		
E	432		24% *ρ*(CCCC) + 14% *ρ*(HCCC) + 14% *π*(CCFeN) + 6% *ρ*(CCCN) + 6% *ρ*(CCNFe)		
A_2_	422	422	42% *ρ*(CCCC) + 14% *ρ*(HCCC) + 12% *π*(CCNC) + 12% *π*(CCFeN)		
E	415	390	41% *ρ*(CCCC) + 14% *ρ*(HCCC) + 14% *π*(CCNC) + 10% *π*(CCFeN)		
E	378	390	43% *ν*(FeN) + 9% *β*(NFeN) + 7% *β*(CCC) + 7% *β*(CNFe) + 7% *π*(NNNFe)	253, 257	225
A_1_	368		27% *ν*(CC*) + 26% *β*(NCC) + 17% *β*(CCC) + 6% *ν*(FeN) + 5% *β*(CNC) + 5% *β*(NFeN)		
E	362		36% *β*(NCC) +17% *ν*(CC*) + 15% *β*(CCC) + 11% *β*(CCN) + 11% *β*(CNC) + 9% *β*(NCC) + 6% *ν*(FeN) + 5% *β*(NFeN)		
A_2_	360		17% *ρ*(CCCC) + 13% *ν*(FeN) + 13% *π*(NNNFe) + 9% *β*(CNFe) + 7% *β*(NFeN) + 5% *π*(CCFeN)		
A_1_	276		44% *ρ*(CCCN) + 19% *π*(CCFeN) + 10% *ρ*(CCNFe) + 6% *β*(NFeN)		
E	266	276	39% *ρ*(CCCN) + 19% *π*(CCFeN) + 13% *ρ*(CCCC) + 13% *ρ*(CCNFe) + 5% *ρ*(CNCC)		
A_1_	255		27% *β*(CNFe) + 18% *β*(CCC) + 10% *β*(NFeN) + 7% *ν*(NC) + 5% *ν*(CC) + 5% *β*(NCC)		
E	229	242	15% *β*(NCC) + 14% *π*(CCFeN) + 11% *β*(CCC) + 11% *β*(CNC) + 11% *ρ*(CCCC) + 9% *β*(NFeN)		
E	208		28% *ν*(FeN) + 9% *β*(CNFe) + 9% *β*(NFeN) + 8% *ρ*(CCCC) + 6% *β*(CNC) + 6% *π*(CCFeN) + 5% *ρ*(CCNFe)		
A_2_	189		17% *β*(CNFe) + 15% *π*(CCFeN) + 12% *ρ*(CCCC) + 11% *ρ*(CCNFe) + 8% *π*(NNNFe) + 6% *ρ*(CNFeN) + 5% *ν*(CC)		
E	177		35% *β*(CCC) + 29% *β*(CNC) + 8% *ν*(FeN) + 6% *ν*(CC) + 5% *β*(NCC)		
A_1_	151		23% *β*(CCC) + 20% *β*(CNC) + 12% *ν*(CC) + 12% *ν*(FeN) + 8% *β*(CNFe) + 7% *β*(NFeN)	148, 145	157
A_1_	130		41% *ρ*(NCCN) + 12% *β*(NFeN) + 12% *π*(CCFeN) + 9% *ρ*(CCCN)	130, 121	127
E	118		29% *ρ*(NCCN) + 14% *π*(CCFeN) + 13% *ρ*(CCCN) + 12% *ρ*(CCNFe) + 7% *ρ*(CCCC) + 5% *π*(NNNFe)		
A_2_	91		30% *π*(CCFeN) + 25% *ρ*(CNFeN) + 18% *ρ*(CCNFe) + 10% *ρ*(CCCC) + 5% *π*(NNNFe)		
E	82		44% *π*(CCFeN) + 28% *π*(CCNC) + 12% *ρ*(CCCC)		
A_2_	59		28% *ρ*(CCNFe) + 26% *π*(NNNFe) + 17% *π*(FeCCN) + 8% *π*(CCFeN) + 5% *ρ*(CCCC)		
E	40		51% *π*(CCFeN) + 11% *ρ*(NCCN) + 10% *ρ*(CCCN) + 9% *π*(NNNFe) + 6% *π*(CCNC)		
A_1_	38		34% *ρ*(CCNFe) + 21% *π*(CCFeN) + 12% *β*(NFeN) + 11% *ρ*(CCCN) + 7% *ρ*(NCCN)		
E	32		51% *ρ*(CCNFe) + 20% *π*(CCFeN) + 5% *π*(CCNC)		

^a^ P.E.D. contribution below 5% is not reported.

^b^ *ν* = stretching, *β* = bending, *ρ* = dihedral angle torsion, and *π* = improper angle torsion.

*The low-frequency region*: Table S3(a) shows the PED analysis for the LS state vibrations, while Table II compares the calculated vibrational modes for the LS state with the available experimental data from Ref. 37. The agreement is very satisfactory both in the low- and high-frequency regions. The Fe-N stretch mode is around 380 cm^−1^ for most Fe(II)-polypyridine complexes.^80^ Examining Table S3(a) shows that indeed, a mode at 368 cm^−1^ dominates this vibration, although other contributions are also present.

For the HS state, as already mentioned, only experimental data in the form of wave packet dynamics in ultrafast pump-probe experiments are available.^8,12,15^ The highest temporal resolution was achieved in Ref. 15, which allowed the observation of three modes at 127, 157, and 225 cm^−1^, with the first being the most prominent and the other two gradually weaker. Table S4(a) shows the PED analysis for the calculated modes of the HS state and Table S4(b) shows the mode frequencies with their assignments.^81^ The identification of the modes appearing as wave packets is still a matter of discussion.^15,17^ However, the 225 cm^−1^ mode can safely be attributed to the Fe–N radial stretch vibration as discussed in Ref. 80. In addition, it is nearly identical for all Fe(II)-polypyridine complexes. Table S4(a) (also reported in Table II) indeed shows that the Fe–N stretch is dominated by modes at 257, 253, and 209 cm^−1^ but also at 121, 114, and 110 cm^−1^. In fact, the computed harmonic frequencies and normal mode analysis of the group of frequencies at 209, 253, and 257 cm^−1^ strongly reveal that the asymmetric character of vibrations in this region are all uniquely assignable to Fe–N asymmetric stretching with a minimal contribution of C–N–Fe OOP bending involving the pyridine groups.

Concerning the mode at 157 cm^−1^, Table S4(b) shows that modes calculated between 190 and 145 cm^−1^ could all contribute. While a definitive assignment cannot be given, it is important to note that none of the calculated modes contains a significant contribution of Fe–N stretch vibrations but are rather dominated by bending modes and improper angle torsions. In detail, doublet bands at 145/148 cm^−1^ refer to the bending modes of OOP nitrogen displacements while the doublet at 188/190 cm^−1^ could safely be assigned to nitrogen in-plane bending modes with a significant displacement of Fe atom. Finally, for the mode at 127 cm^−1^, the 130 to 110 cm^−1^ region shows that the Fe–N stretch mode is dominant, although bending and torsional contributions are also present. In fact, the computed harmonic frequencies reveal that normal modes at 110, 114, and 121 cm^−1^ do actually refer to Fe–N symmetric stretch modes with a partial contribution of out-of-plane (OOP) bending involving C–N–Fe and C–C–C groups. In summary, we can safely attribute the 127 and 225 cm^−1^ vibrations to predominantly Fe–N stretch modes, while the 157 cm^−1^ vibration is dominated by bending and torsional modes. These assignments are reported in Table II.

*The high frequency region*: The modes spanning from 400 to 3000 cm^−1^ are predominantly due to the organic ligand (Table S2(b)) and the major changes due to the electronic state may be revealed in the CH stretching region and around 1600, 1400, 1300, 700, and below 600 cm^−1^. This is highlighted in Tables S5 and S6 for both states where the calculated vibrational frequencies (in cm^−1^), the corresponding IR band intensity (in km mol^−1^) values and symmetry are provided, and the spectra are shown in Fig. S5. In Table S5, the A_1_ symmetry vibrations of the LS state are also included. Although these modes have no IR activity in the D_3_ symmetry group, the symmetry breakdown occurring in the HS state is expected to make them IR active and to lift the degeneracy of doubly degenerate modes. Thus, in principle, the vibrational spectrum of the HS state is expected to be more complex than that of the LS state.

The CH stretch region is expected to reflect significant changes of the vibrational structure between the two electronic states. The calculated LS and HS CH stretch frequencies and occasionally, the IR band intensity for the most intense bands is shown in Tables S4 and S5 for the LS and HS states.

From these results, one may easily foresee that several bands of each electronic state are actually expected to overlap. This conclusion is schematically represented in Table S5 reporting the frequencies of the peaks which overlap. Relying on these computations, the most intense CH stretch bands would be produced by the LS state around 3150 cm^−1^ while the lowest frequency cluster of bands would be evidence of the HS state around 3120 cm^−1^.

Employing the same approach, let us consider the other bands of the spectra of the two electronic states. As one may note in Fig. S5, the modes in the intermediate region of the HS state for which the three bands centered at 1590 cm^−1^ and those at 1580 cm^−1^ produce intense components while the lower intensity band at 1565 cm^−1^ is the result of the overlap of the three bands marked as ( $\begin{array}{c} {}*\\ {}* \end{array}$) in Table S6. The same reasoning can be employed for the other typical bands, namely, the multiplet at 1400 cm^−1^ (CC stretching, CH and ring bend modes), the band at 1300 cm^−1^ (CN and CC stretch modes) and the bands in the 600–800 cm^−1^ range and those below 600 cm^−1^, mainly ring modes and CH rocking modes, described in detail in Table S6.

In this way, for the LS ground state it is evident that the 1600 cm^−1^ closely lying modes would produce a single band whose intensity is largely due to the E symmetry vibrations and the lower intensity band around 1560 cm^−1^ is due to the overlap between the E and A_2_ modes in this range. This region is very different in the HS state where the LS vibrational modes are now replaced by a clear triplet of mode frequencies around 1590, 1580, and 1550 cm^−1^. The same applies to the bands between 1470 and 1432 cm^−1^, and the next bands are the three medium-weak intensity bands in the 1300–1200 cm^−1^ range and in the region of 700 cm^−1^. The above discussion, along with Fig. S5, provides a recipe for the identification of the distortions in the HS state by means of time-resolved IR or Raman spectroscopy, as recently reported in Ref. 45.

## IV. CONCLUSIONS

We have investigated the electronic and molecular structure of the low-spin ground state and the high-spin excited state of the prototypical [Fe(bpy)_3_]^2+^ molecule, using the PBE^*^ functional, modeling the solvent interaction via C-PCM method. The calculated molecular structure of the HS state points to a nonequivalent elongation of the six Fe–N bonds in line with previous theoretical predictions,^28,31^ and more recent computational reports,^22,34^ as well as to a distortion of the bpy ligands, leading to a symmetry lowering of the system from D_3_ in the LS state to C_1_. The results of the calculations are benchmarked against the available experimental observables, namely, the ultraviolet-visible absorption spectra, the steady-state and transient Fe K-edge XANES spectra and ultraviolet circular dichroism spectra for both the LS and HS states. Bearing in mind that we are dealing with the most probable configuration of a rather floppy molecule, the simulations do provide good agreement with the experiment, correlating these very diverse observables to one set of structural parameters. The observed symmetry reduction along the LS→HS transition is expected to cause the mixing of electronic states and therefore the relatively short lifetime of the HS spin state.

Finally, the stationary points of the potential energy surface were characterized as local minima by calculating harmonic vibration frequencies. The resulting calculated low-frequency vibrational modes are in good agreement with the data from pump-probe experiments and allow us to identify the modes that appear as wave packets in the HS state. However, these are not the modes that cause the asymmetric deformation in the HS. We computed the frequencies and IR absorption spectra from the QM harmonic force field for the LS and HS states, which show substantial differences between them, especially in the mid- to high-frequency region, thus providing a tool for further investigations of the distortions in the HS state using transient IR spectroscopy or time-resolved Raman spectroscopy.

## Data Availability

The data that support the findings of this study are available from the corresponding authors upon reasonable request.
